# Network analysis to estimate central insomnia symptoms among daytime workers at-risk for insomnia

**DOI:** 10.1038/s41598-023-43802-7

**Published:** 2023-09-29

**Authors:** Yuta Takano, Rui Ibata, Norihito Nakano, Yuji Sakano

**Affiliations:** 1 Department of Somnology, Tokyo Medical University, 5-10-10 Yoyogi, Shibuya-ku, Tokyo, 151-0053 Japan; 2 Japan Somnology Center, Neuropsychiatric Research Institute, Tokyo, Japan; 3 Graduate School of Psychological Science, Health Sciences University of Hokkaido, Hokkaido, Japan; 4 Goryokai Medical Corporation, Hokkaido, Japan; 5 School of Psychological Science, Health Sciences University of Hokkaido, Hokkaido, Japan; 6 Sapporo CBT & EAP Center, Goryokai Medical Corporation, Hokkaido, Japan

**Keywords:** Psychology, Health occupations

## Abstract

Although insomnia complaints are associated with mental health problems and reduced work productivity, the central insomnia symptoms in workers at-risk for insomnia remain unclear. This study aimed to identify the central insomnia symptoms among daytime workers at risk for insomnia. The participants were 881 Japanese daytime workers at-risk for insomnia with a mean age of 49.33 ± 9.92 years. At-risk for insomnia was defined as an Athens Insomnia Scale score of six or higher. The Athens Insomnia Scale was used as a screening for at-risk insomnia because it has higher sensitivity and specificity than other insomnia screening scales. The Insomnia Severity Index is recommended as a mechanism of insomnia and an outcome measure; therefore, a network analysis was conducted with the seven items of the Insomnia Severity Index. The important variables in the connections between insomnia symptoms were estimated from centrality indices, which were interpretable only for strength. The strength value results suggest that difficulty staying asleep and worry about sleep problems were the central insomnia symptoms. The connections were stronger for difficulty staying asleep and problem waking up too early, difficulty staying asleep and difficulty falling asleep, and interference with daytime functions and noticeable to others. Worry about sleep problems was strongly associated with variables other than nocturnal insomnia symptoms. Therefore, difficulty staying asleep and worry about sleep problems are important variables in daytime workers at-risk for insomnia and are key points for improvement or exacerbation of insomnia symptoms.

## Introduction

An epidemiological study on insomnia in Japan estimated the probability of insomnia disorder to be 12.2% in men and 14.6% in women^1^. The prevalence of difficulty falling asleep, difficulty staying asleep, and early morning awakenings are estimated to be 8.3%, 5.8%, and 5.8% in men and 11.0%, 8.1%, and 7.4% in women, respectively^1^. Insomnia complaints have been associated with mental health problems and reduced work productivity^2,3^. Therefore, it is important to focus on insomnia complaints to manage daytime workers’ healthcare.

The network analysis method is often used to estimate the interactions among symptoms. In network analysis, partial correlation coefficients between variables are calculated and visualized as a network diagram^4^. It is similar to structural equation modeling (SEM), which is used for visualizing associations between variables. However, it is different from SEM. In network analysis, the centrality index can identify variables that have a significant influence on the model^5^. Intervening in the central symptoms of the network may enable rapid recovery from psychological symptoms^5^. In other words, central symptoms are specific symptoms that have multiple symptom-to-symptom associations and are expected to be related to alleviation or exacerbation of the severity of the symptoms. The use of network analysis in this study is an excellent choice because it helps identify the intervention targets for rapid recovery of insomnia symptoms.

Network analyses have been conducted in several studies on insomnia symptoms. Results of a network analysis of the five-factor model personality traits and insomnia symptoms in the general population in Netherlands showed that sleep dissatisfaction is meaningful in the link between insomnia symptoms and personality traits^6^. A study using network analysis for predicting first-onset major depressive disorder showed that difficulty falling asleep was a predictor, even when depressive symptoms were controlled^7^. Additionally, two studies have performed network analysis of insomnia, depressive, and anxiety symptoms. In a study of adults aged over 18, difficulty staying asleep was estimated to be the central symptom in the symptom-to-symptom association^8^. In another study of adults aged over 18 who met the DSM-5^9^ criteria for insomnia disorder and the Sleep Condition Indicator^10^ screening for insomnia symptoms, a sense of uncontrolled worry was estimated as a central symptom^11^. A contributing factor to the differences in these network analyses may be whether the participants are from the general population, including healthy individuals, or whether they are only individuals with insomnia symptoms.

Only one study has conducted network analysis of insomnia symptoms alone. Bai et al.^12^ used the Insomnia Severity Index (ISI)^13^ to estimate central insomnia symptoms in mental health professionals during the COVID-19 pandemic. They reported that daytime dysfunction was the central symptom and that no gender differences were found in the symptom-to-symptom association of insomnia symptoms. However, there are several problems with the findings. For example, they did not establish the criteria for insomnia, included individuals who did not complain of insomnia, and they did not exclude individuals who worked night shifts. These factors skew the results. Insomnia in shift workers is often a circadian rhythm misalignment due to work schedules^14^. In general, insomnia is associated with sleep-related cognitive processes^15^. That is, the causes and maintenance processes of insomnia differ between shift workers and daytime workers. Therefore, shift workers must be excluded. Also, given the possibility that the results of the network analysis may differ depending on whether or not the participants have insomnia complaints^8,11^, it is necessary to study individuals with insomnia complaints to determine the clinically meaningful central symptoms of insomnia.

There are several self-report scales that screen for insomnia symptoms and determine the effectiveness of treatment for insomnia. A meta-analysis of diagnostic accuracy showed that although the Athens Insomnia Scale (AIS)^16^ did not indicate statistical differences in diagnostic accuracy compared to other scales, it had the highest values for both sensitivity and specificity^17^. Therefore, the AIS was used for screening for insomnia in this study, and individuals above the cut-off point of the AIS were defined as at-risk for insomnia. ISI is recommended for use in studies of the mechanism/evaluation of insomnia disorder^18^. Therefore, ISI was used for network analysis. This study aimed to estimate the central insomnia symptoms among daytime workers at-risk for insomnia.

## Methods

### Participants and procedure

The present study was conducted as part of an Internet-based cross-sectional survey of insomnia and work productivity among Japanese daytime workers. Part of the data pooled in this survey has already been published^3^. However, Takano et al.^3^ did not examine the associations between insomnia symptoms. The survey period was from November 16–18, 2021. Of the 3438 participants who provided informed consent to participate in the previous research, 881 (702 men and 179 women) met all eligibility criteria for this study. Informed consent was obtained from all subjects and/or their legal guardian(s) for this study. The eligibility criteria included working eight hours per day for five days per week (full-time workers), no night shifts, no applicability to inattention detection items, no incomplete responses, no other gender, and a score of six or higher on the Japanese version of the AIS^19^. Inattention detection items are questions (Directed Questions Scale) that identify those who respond to a question without reading it^20^.

**Figure 1:**
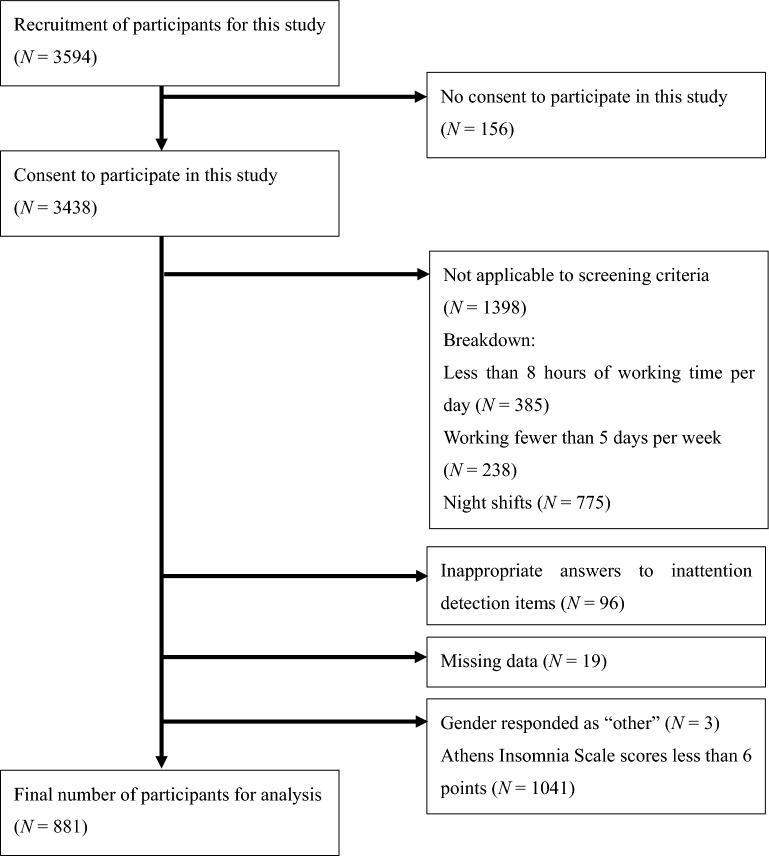
Flowchart of the participant selection process in this study.

The process for selecting the participants for this study is shown in Fig. 1.

All procedures performed in the study were in accordance with the ethical standards of the institutional and/or national research committee and with the 1964 Helsinki Declaration and its later amendments or comparable ethical standards. The present study was approved by the Ethics Board of the School of Psychological Science, Health Sciences University of Hokkaido, Japan (Approval number: 22004).

### Measurements

This study obtained demographic data of all participants, including age, gender, body mass index (BMI), and occupation.

The Japanese version of the AIS is a self-report scale for assessing insomnia symptoms^19^. The AIS is an eight-item questionnaire with a four-point Likert scale. The Japanese version of the AIS has been confirmed to have concurrent validity with the Japanese version of the ISI (*r* = 0.85)^19^. Individuals with scores of six or higher on the Japanese version of the AIS^17^ were defined as being at-risk for insomnia in this study.

The Japanese version of the ISI is another self-report scale for assessing insomnia symptoms^21^. The ISI is a seven-item questionnaire that uses a five-point Likert scale to rate responses and focuses on nocturnal sleep disturbances such as sleep-onset problems (ISI_1), sleep-maintenance problem (ISI_2), early morning problems (ISI_3), sleep dissatisfaction (ISI_4) , daytime dysfunction such as interference with daily functioning (ISI_5) and noticeability of impairment due to the sleep disturbance (ISI_6), and emotional distress due to sleep disturbance (ISI_7)^13^.

### Statistical analysis

R 4.2.2 was used for the statistical analysis. The R packages *qgraph* (Version: 1.9.2), *bootnet* (Version: 1.5), and *NetworkComparisonTest* (NCT, Version: 2.2.1) were used for the network analysis. The visualized network diagram consisted of variables (nodes) connected by lines (edges). In this study, each ISI item was a node. The edges of the network are polychoric correlations of the seven items of the ISI. This study estimated the Gaussian graphical model (GGM) network from the least absolute shrinkage and selection operator and extended Bayesian information criteria. In the GGM, edges can be interpreted as partial correlation coefficients^4^. In the network diagram, the relationship between nodes was represented by the width of the edges and thickness of the color. The stability of the centrality indices was confirmed by the correlation-stability (*CS*) coefficient. A *CS* coefficient of 0.25 or less is considered unstable; a *CS* coefficient of 0.5 or more is recommended^4^. Case-dropping bootstrapping with 2500 samples was used to estimate *CS* coefficients. This study used three centrality indices—strength, betweenness, and closeness^22^. Strength was estimated as the strength of a node’s direct connection to the network. Betweenness was estimated from the concept of the shortest path length connecting any two nodes, and nodes with high distance centrality between them were considered central in connecting other symptoms. Closeness was estimated as how strongly a node is indirectly connected to the network.

Differences in the estimated network of ISI scores between male and female participants were examined using network structure, global strength, and centrality indices. The *p*-values were adjusted using the Bonferroni method. Network structure is the maximum difference in pairwise edges between the male and female networks. Global strength is the sum of all edges between the male and female networks.

Welch’s *t*-test was used to determine whether there were sex differences in age, BMI, ISI total scores, and each ISI item score. The effect size was calculated as Hedges’ *g*. The R package *compute.es* (Version: 0.2.5) was used for the effect size.

## Results

### Participant characteristics

Participants’ demographic data and ISI scores are shown in Table 1. Participant occupation categories were as follows: administrative and managerial (25.1%); professional and engineering (22.8%); clerical (27.5%); sales (5.2%); service (5.3%); security (0.7%); agriculture, forestry, or fishery (0.2%); manufacturing process (5.6%); transport and machine operation (1.0%); construction and mining (1.3%); carrying, cleaning, or packaging (1.1%); and workers not classified by occupation (4.2%).

**Table 1:** Participants’ demographic data and ISI scores.

	Overall (*N* = 881)		Male (*N* = 702)		Female (*N* = 179)		*t*	Hedges’ *g*
	*M*	*SD*	*M*	*SD*	*M*	*SD*		
Age (years)	49.33	9.92	51.39	8.59	41.28	10.69	− 11.72*	1.12
BMI	23.07	3.86	23.74	3.70	20.43	3.33	− 11.56*	0.91
ISI total score	11.74	4.64	11.82	4.53	11.43	5.03	− 0.95	0.08
Difficulty falling asleep (ISI_1)	1.29	1.03	1.25	1.03	1.42	1.01	2.03*	− 0.17
Difficulty staying asleep (ISI_2)	1.50	1.00	1.50	0.98	1.48	1.08	− 0.24	0.02
Problem waking up too early (ISI_3)	1.60	1.04	1.67	1.02	1.32	1.09	− 3.86*	0.34
Dissatisfaction (ISI_4)	2.68	0.80	2.70	0.78	2.60	0.90	− 1.29	0.12
Interference with daytime functions (ISI_5)	1.57	0.86	1.58	0.83	1.55	0.97	− 0.37	0.03
Noticeable to others (ISI_6)	1.33	0.93	1.34	0.92	1.27	0.99	− 0.89	0.07
Worry/Distress (ISI_7)	1.79	0.96	1.78	0.95	1.79	1.01	0.03	− 0.01

*M* Mean, *SD* Standard deviation, *BMI* Body mass index, *ISI* Insomnia Severity Index.

*p < 0.05.

### Estimation of network structure

**Figure 2:**
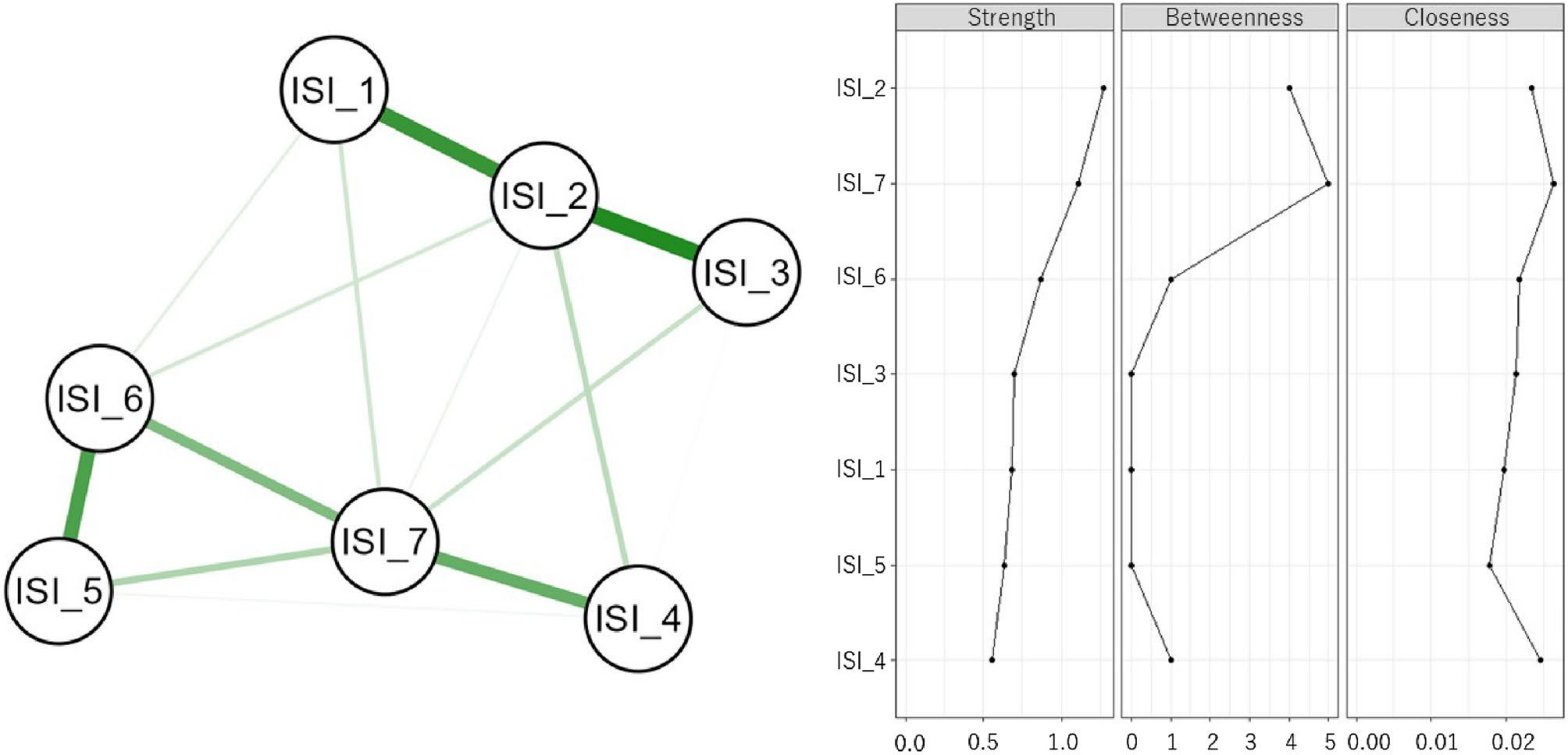
Results of the network analysis. The left side presents the network diagram of the Insomnia Severity Index (ISI) results for the study participants. The green edges are positively correlated and the red edges are negatively correlated. The right side displays the resulting centrality indices, ordered by strength. Abbreviations: Difficulty falling asleep (ISI_1), Difficulty staying asleep (ISI_2), Problem waking up too early (ISI_3), Dissatisfaction (ISI_4), Interference with daytime functions (ISI_5), Noticeable to others (ISI_6), and Worry/Distress (ISI_7).

**Figure 3:**
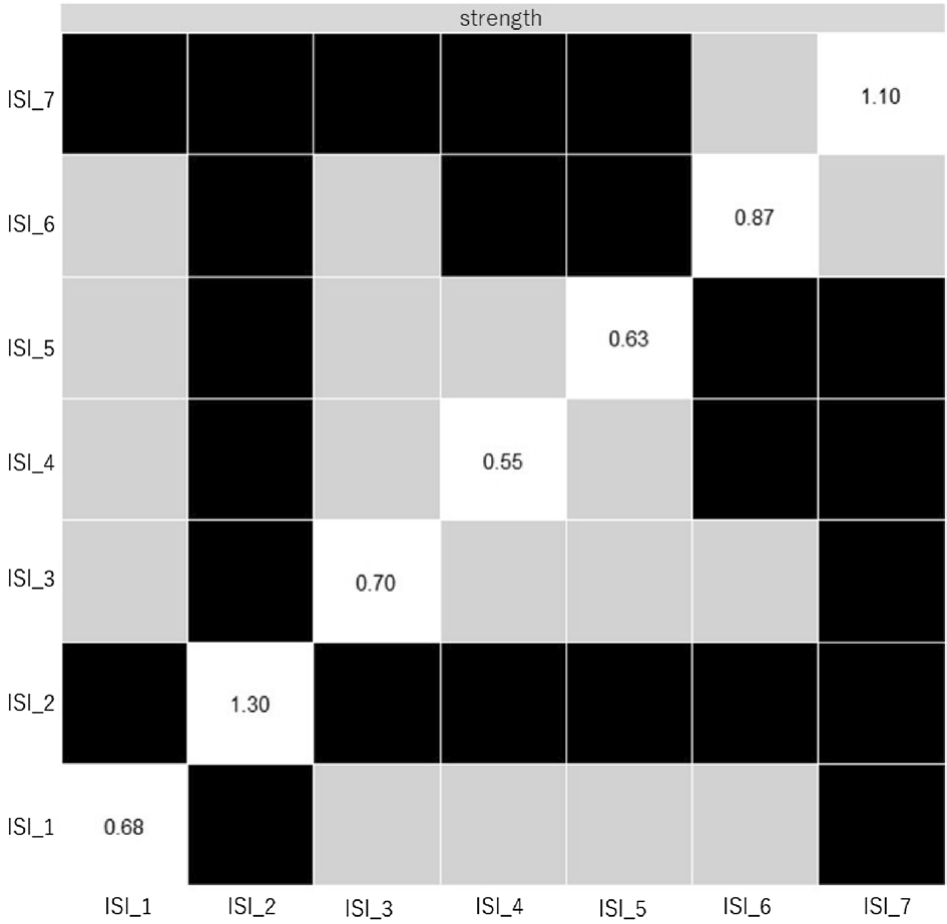
Bootstrapped difference tests (α = 0.05) results between the node strength of the variables in this study. The black boxes indicate a significant difference between the nodes, the gray boxes indicate no significant difference between the nodes. Abbreviations: Difficulty falling asleep (ISI_1), Difficulty staying asleep (ISI_2), Problem waking up too early (ISI_3), Dissatisfaction (ISI_4), Interference with daytime functions (ISI_5), Noticeable to others (ISI_6), and Worry/Distress (ISI_7).

The network diagram based on each ISI item and the three centrality indices are shown in Fig. 2. *CS* coefficients indicated node strength (*CS* = 0.75), betweenness (*CS* = 0.21), and closeness (*CS* = 0.28). Strength was the only centrality index with a *CS* coefficient above 0.5. The centrality index of closeness was above 0.25 but below 0.5; the centrality index of betweenness was below 0.25. In other words, strength was stable and interpretable, but closeness and betweenness should be interpreted cautiously. A comparison of strength values is shown in Fig. 3. Difficulty staying asleep (ISI_2) was significantly higher than all other variables. Worry about sleep problems (ISI_7) was not significantly different from noticeable to others (ISI_6), but worry about sleep problems (ISI_7) was significantly higher than difficulty falling asleep (ISI_1) and problem waking up too early (ISI_3). In contrast, noticeable to others (ISI_6) was not significantly different from difficulty falling asleep (ISI_1) and problem waking up too early (ISI_3).

**Figure 4:**
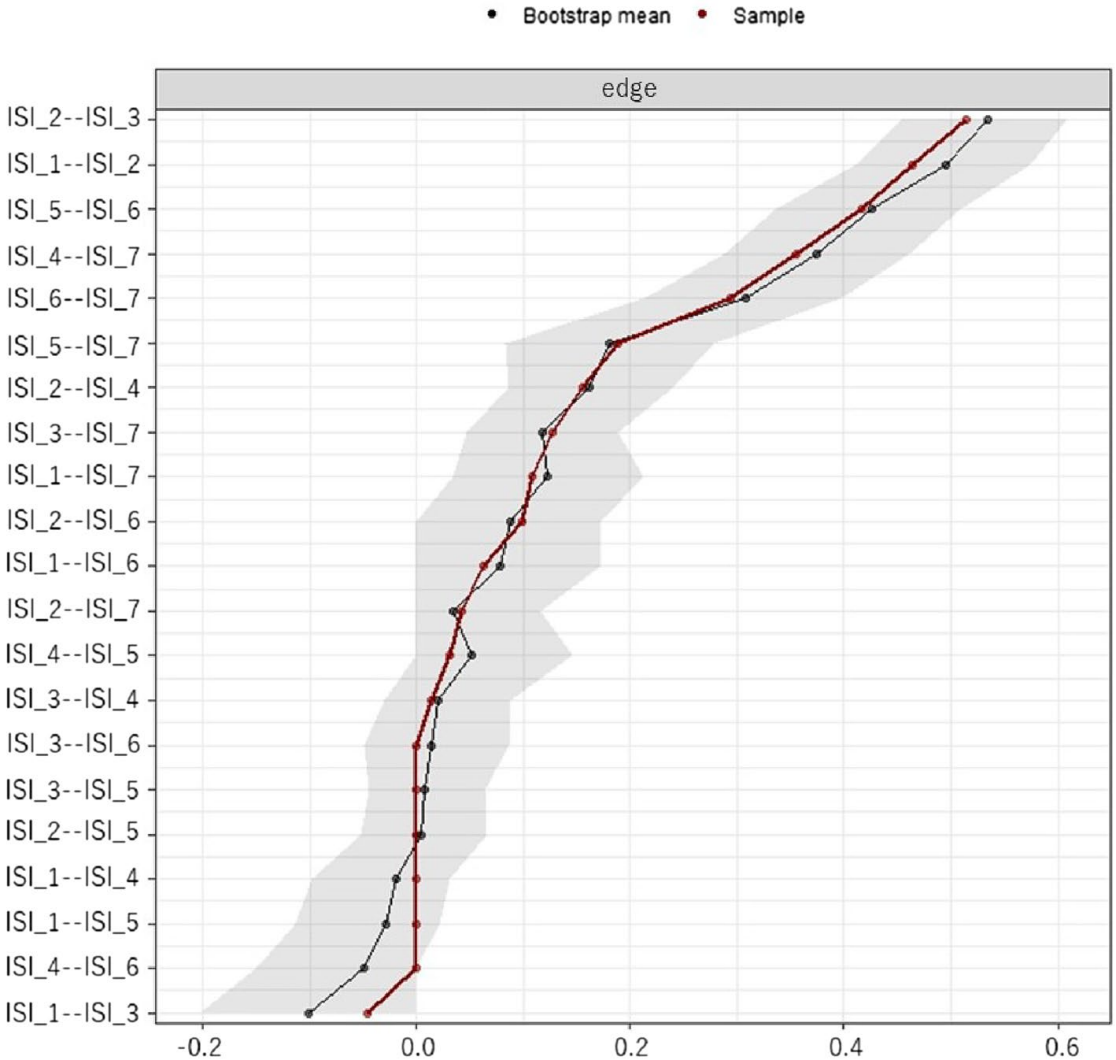
Bootstrap confidence intervals were estimated to indicate the magnitude of the edges-weighted between variables in the network structure. The gray ranges are the bootstrap confidence interval. The dots are plotted polychoric correlation coefficient values. Abbreviations: Difficulty falling asleep (ISI_1), Difficulty staying asleep (ISI_2), Problem waking up too early (ISI_3), Dissatisfaction (ISI_4), Interference with daytime functions (ISI_5), Noticeable to others (ISI_6), and Worry/Distress (ISI_7).

Bootstrap confidence intervals were estimated to indicate the magnitude of the edges-weighted between variables in the network structure (Fig. 4). The relationships among variables in the network structure were strongly related in the order of difficulty staying asleep (ISI_2) and problem waking up too early (ISI_3) (polychoric correlation coefficient = 0.53, 95% confidence interval (CI) [0.45, 0.61]), difficulty staying asleep (ISI_2) and difficulty falling asleep (ISI_1) (polychoric correlation coefficient = 0.49, 95% CI [0.40, 0.57]), and interference with daytime functions (ISI_5) and noticeable to others (ISI_6) (polychoric correlation coefficient = 0.43, 95% CI [0.34, 0.51]). Worry/distress (ISI_7) was associated with variables other than nocturnal sleep problems (ISI_4: polychoric correlation coefficient = 0.37, 95% CI [0.28, 0.46]; ISI_6: polychoric correlation coefficient = 0.31, 95% CI [0.22, 0.39]; ISI_5: polychoric correlation coefficient = 0.18, 95% CI [0.09, 0.27]).

### Comparison of network structure by gender

The male and female ISI network structures did not demonstrate significant differences in terms of network structure (*p* = 0.17), global strength (*p* = 0.53), and centrality index values of strength (all *p* > 0.05).

## Discussion

This study’s findings suggest that difficulty staying asleep and worry about sleep problems are central insomnia symptoms among workers at-risk for insomnia. Moreover, difficulty staying asleep and worry about sleep problems were more strongly connected with other insomnia symptoms. Thus, the strength values were higher than other insomnia symptoms. Changes in difficulty staying asleep and/or worry about sleep problems may affect other insomnia symptoms, either improving or exacerbating them.

The inclusion of worry about sleep problems is consistent with the cognitive model of insomnia^23^. A meta-analysis of cognitive behavioral therapy for insomnia found that it moderately improved worry and difficulty staying asleep^24,25^. CBT-I has been shown to be effective in improving insomnia symptoms in daytime workers^26^. A network analysis comparing the pathways of improvement in insomnia symptoms between Internet-delivered behavior and cognitive therapies showed that behavior therapy improved sleep efficiency, difficulty staying asleep, and dissatisfaction with sleep patterns, while cognitive therapy improved difficulty falling asleep, problems waking up too early, interference with daytime functions, and worry about sleep problems^27^. In Blanken et al.’s^27^ network analysis of treatment processes, behavior therapy showed a direct association with difficulty staying asleep at four weeks compared to cognitive therapy, but fundamentally extended to an improvement in other insomnia symptoms via increased sleep efficiency. Cognitive therapy showed a direct association with worry about sleep problems at 10 weeks post-treatment compared to behavior therapy^27^. The results for face-to-face behavior therapy and cognitive therapy were consistent with those of Blanken et al.^27^, except that worry about sleep problems changed during the process of treatment^28^. It is hypothesized that if the central symptoms identified in the network analysis can be addressed, overall symptoms could improve more quickly^5^. The results of this study revealed that the central symptoms of insomnia were difficulty staying asleep and worry about sleep problems. Previous studies have found that these two symptoms changed during the course of treatment^27,28^. Although to our knowledge no studies have focused only on difficulty staying asleep and worry about sleep problems, changes in these two symptoms may contribute significantly to changes in overall insomnia symptoms. Future studies should examine whether there is a difference in difficulty staying asleep and worry about sleep problems between individuals who experienced alleviation of insomnia symptoms at the end of CBT-I and those who did not. This could help determine whether these two symptoms are robust as central symptoms.

Bai et al.^12^ estimated that daytime dysfunction was a central insomnia symptom. However, our study estimated that the daytime dysfunction was not enough to change the whole network. The independent diagnostic criteria for insomnia disorder include nocturnal sleep problems and daytime dysfunctions^29^. Furthermore, insomnia symptoms have been found to be more persistent over time in the nocturnal sleep problems and psychological distress and/or daytime dysfunction group than in the nocturnal sleep problems, psychological distress, or dysfunction alone group^30^. In individuals who complain of insomnia, difficulty staying asleep and worry about sleep problems are important factors in intervention and assessment. Specifically, the assessment may be important for the early detection of risk for severe insomnia. Moreover, early interventions for people who experience difficulty staying asleep and/or worry about sleep problems may prevent other insomnia symptoms from worsening and, consequently, may decrease the severity of insomnia.

A worldwide epidemiological study during the COVID-19 pandemic estimated that 17.4% of people were likely to have insomnia disorder, and this estimate was 7.9% for people in Japan^31^. Moreover, an epidemiological study in Japan conducted before the COVID-19 pandemic found that 12.2% of men and 14.6% of women had a probability of insomnia disorder^1^. In other words, the probability of insomnia disorder may not have increased much during the COVID-19 pandemic in Japan. Therefore, the results of this study might be applicable after the COVID-19 pandemic.

This study has several limitations. First, because the *CS* coefficients for closeness and betweenness were below 0.5, the results were interpreted based on strength. However, the order of the nodes with the highest scores of the centrality index of strength does not fully match that of closeness and betweenness. Therefore, central symptoms in this study reflect only the degree of connection with other variables in the network. Second, due to a cross-sectional design, causality could not be determined. In other words, it is unclear whether difficulty staying asleep and worry about sleep problems affect other symptoms in the network or whether other nodes in the network affect difficulty staying asleep and worry about sleep problems. It is also possible that characteristics of the participants’ insomnia symptoms at the time of the survey may have been represented. Third, as the participants for this study were recruited through an Internet survey, sampling bias was present; however, the quality of responses was ensured by using items to detect participants who did not read the questionnaire. Fourth, the participants were middle-aged, as the mean age was 49.33 years. Insomnia symptoms were affected by age, as difficulty falling asleep is more frequent in younger than in middle-aged persons, while in middle-age, difficulty staying asleep is more frequent than difficulty falling asleep^32^. The results of our study may be limited to insomnia symptoms in middle-aged daytime workers. Fifth, this study gathered data during the COVID-19 pandemic. This study has not been able to separate essential workers such as medical personnel from other workers. In other words, the study has not been able to consider the stress level of occupations due to the COVID-19 pandemic. Sixth, this study was targeted only to daytime workers at the time of recruiting the participants, which may have caused a selection bias.

Despite these limitations, difficulty staying asleep and worry about sleep problems may be central insomnia symptoms for daytime workers at-risk for insomnia. As insomnia complaints are associated with mental health problems and reduce work productivity, identifying central insomnia symptoms may predict the occurrence of health problems in workers.

## Data Availability

The research data used in this study are available only for rational reasons by contacting the corresponding author.
